# Deciphering the Mechanism of Wogonin, a Natural Flavonoid, on the Proliferation of Pulmonary Arterial Smooth Muscle Cells by Integrating Network Pharmacology and In Vitro Validation

**DOI:** 10.3390/cimb45010037

**Published:** 2023-01-08

**Authors:** Lidan Cui, Zuomei Zeng, Xinyue Wang, Tianyi Yuan, Can Wang, Dianlong Liu, Jian Guo, Yucai Chen

**Affiliations:** 1 School of Traditional Chinese Medicine, Beijing University of Chinese Medicine, Beijing 100029, China; 2 State Key Laboratory of Bioactive Substance and Function of Natural Medicines, Institute of Materia Medica, Chinese Academy of Medical Sciences and Peking Union Medical College, Beijing 100050, China; 3 School of Chinese Pharmacy, Beijing University of Chinese Medicine, Beijing 100029, China

**Keywords:** network pharmacology, pulmonary hypertension, cell proliferation, phenotypic switch, HIF-1α

## Abstract

Wogonin is one of the main active components of *Scutellaria baicalensis*, which has anti-inflammatory, anti-angiogenesis, and anti-fibrosis effects. Nevertheless, the effect of wogonin on pulmonary hypertension (PH) still lacks systematic research. This study aims to elucidate the potential mechanism of wogonin against PH through network pharmacology and further verify it through biological experiments in pulmonary arterial smooth muscle cells (PASMCs). The potential targets and pathways of wogonin against PH were predicted and analyzed by network pharmacology methods and molecular docking technology. Subsequently, the proliferation of PASMCs was induced by platelet-derived growth factor-BB (PDGF-BB). Cell viability and migration ability were examined. The method of Western blot was adopted to analyze the changes in related signaling pathways. Forty potential targets related to the effect of wogonin against PH were obtained. Based on the protein–protein interaction (PPI) network, gene-ontology (GO) and Kyoto encyclopedia of genes and genomes (KEGG) enrichment, and molecular docking, it was shown that the effect of wogonin against PH is closely related to the proliferation of PASMCs and the hypoxia-inducible factor-1α (HIF-1α) pathway. A variety of results from biological experiments verified that wogonin can effectively inhibit the proliferation, migration, and phenotypic transformation of PDGF-BB-mediated PASMCs. In addition, the anti-proliferation effect of wogonin may be achieved by regulating HIF-1/ NADPH oxidase 4 (NOX4) pathway.

## 1. Introduction

Pulmonary hypertension (PH) is a progressive disease characterized by increased vasoconstrictor tone at the early stage, leading to small pulmonary artery remodeling, elevated pulmonary vascular resistance, and right heart failure [1]. The exact etiologies of PH are likely to vary with the underlying pathogenic or genetic cause, which are ultimately attributed to concentric medial thickening of small arterioles. The prognosis of PH is very poor. Although prostacyclin and its analogs, endothelin receptor antagonists, phosphodiesterase-5 inhibitors, and other drugs have been used in the clinical treatment of PH, the 1-year mortality of PH patients is still as high as 15%, and the 3-year survival rate of idiopathic pulmonary artery hypertension (IPAH) patients is as low as 35% [2].

The process of pulmonary vascular remodeling involves abnormal proliferation of pulmonary arterial smooth muscle cells (PASMCs), the injury of pulmonary arterial endothelial cells (PAECs), and deposition of extravascular collagen [3]. The mechanism of pulmonary vascular remodeling is very complex, involving many systems such as nerve, endocrine, immune inflammation, and so on. Recent studies have shown that oxidative stress-related signaling pathways may play an important role in pulmonary vascular remodeling [4]. Antioxidant therapy has been proven to effectively inhibit pulmonary vascular remodeling and reduce and improve the symptoms of PH [5]. 

Natural products are one of the major sources of modern innovative medicines. With the continuous progress of modern pharmacology and molecular biology techniques, natural products have attracted more attention because of their advantages of low side effects and multiple targets. A variety of compounds from multiple natural sources, including tanshinone IIA and ligustrazine, are being tried and applied in the treatment of PH at preclinical and clinical stages [6,7,8]. Wogonin, a flavonoid extracted from the dried roots of *Scutellaria baicalensis* Georgi, is one of the major active components of the traditional Chinese medicine *Scutellaria baicalensis*. Recent studies have indicated that wogonin has great potential in the treatment of cardiovascular diseases including diabetic cardiomyopathy, cardiac hypertrophy, and dilating blood vessels [9,10,11]. Nevertheless, the effect of wogonin on pulmonary circulation-related diseases still lacks systematic research. 

**Figure 1:**
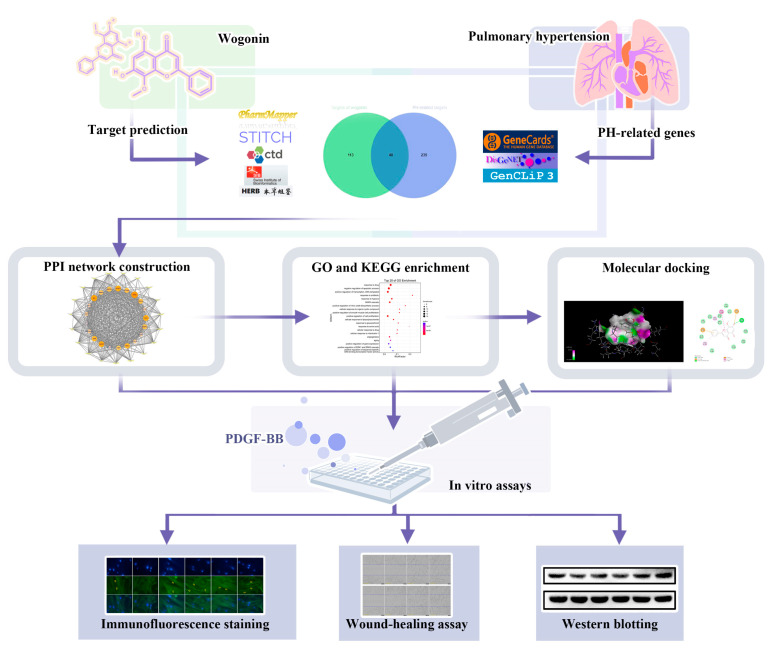
The experimental flow of this study. PPI: protein–protein interaction; GO: gene-ontology; KEGG: kyoto encyclopedia of genes and genomes; PDGF-BB: platelet-derived growth factor-BB.

Based on the above status, in the present study, the approaches of network pharmacology and molecular docking were employed to analyze the key targets and signaling pathways of wogonin in the treatment of PH. Based on the results, experiments in vitro were performed to explore the effects of wogonin on PASMC proliferation, and the previously predicted potential mechanism of wogonin was experimentally verified at the molecular level (Figure 1).

## 2. Materials and Methods

### 2.1. Reagents and Materials

Wogonin, bosentan, and PDGF-BB were purchased from MedChemExpress (Israel Shekel). High-glucose Dulbecco’s Modified Eagle’s Medium (DMEM) and fetal bovine serum (FBS) were purchased from Gibco (Thermo Fisher Scientific Inc., Waltham, MA, USA). Phalloidin and Hochest 33342 were purchased from Abcam (Cambridge, UK). Primary antibodies specific for vascular cell adhesion molecule 1 (VCAM-1), intercellular adhesion molecule 1 (ICAM-1), and HIF-1α were procured from ProteinTech (Chicago, IL, USA). Primary antibodies against NOX4, α-smooth muscle actin (α-SMA), and calponin 1 were purchased from Abcam (Cambridge, UK). The Cell Counting Kit-8 (CCK-8) assay was purchased from Dojindo Laboratories (Kumamoto, Japan). Other reagents and chemicals were all analytical grade.

### 2.2. Collection of Target Genes

To collect a comprehensive list of targets of wogonin, four databases were searched: PharmMapper Server (http://www.lilab-ecust.cn/pharmmapper/ (accessed on 18 August 2021), fit score >3) [12], STITCH (http://stitch.embl.de/ (accessed on 18 August 2021)) [13], Comparative Toxicogenomics Database (http://ctdbase.org/) [14], SwissTargetPrediction (http://www.swisstargetprediction.ch (accessed on 18 August 2021), score > 0.1) [15], and HERB database (http://herb.ac.cn/ (accessed on 18 August 2021)) [16]. 

The PH-related genes were identified using the Genecards database (https://www.genecards.org/ (accessed on 19 August 2021), score > 10) [17], the DisGeNET platform (http://www.disgenet.org/ (accessed on 19 August 2021)) [18], and GenCLiP 3.0 online tool (http://ci.smu.edu.cn/ (accessed on 19 August 2021), hit score > 5) [19]. The search term was set as “pulmonary hypertension”, and reproducible targets were removed. 

The intersection of targets of wogonin and PH-related proteins was considered potential therapeutic targets of wogonin against PH.

### 2.3. PPI Network Construction

The gene list of therapeutic targets of wogonin against PH was uploaded to the STRING (cn.string-db.org) database to construct the PPI network. The species is set as “Homo sapiens”. The obtained PPI network is imported into Cytoscape 3.8.2 software for visualization. Using the NetworkAnalyzer plugin, the topological parameters of the network were analyzed. The larger the degree value of a node in a network, the higher its core properties in the network are represented.

### 2.4. GO and KEGG Enrichment

The Database for Annotation, Visualization and Integrated Discovery (DAVID) v6.8 (https://david.abcc.ncifcrf.gov/ (accessed on 1 September 2021)) was employed for enrichment analysis of therapeutic targets of wogonin against PH [20]. The top 20 terms of biological process (BP), molecular function (MF), and KEGG pathways with the highest enrichment were obtained. The obtained GO and KEGG terms are imported into the Omicshare platform (https://www.omicshare.com/ (accessed on 1 September 2021)) for visualization.

### 2.5. Molecular Docking

The 3D structure of wogonin (ID: 5281703) was downloaded from the PubChem database. The crystal structure of the PDGF receptor-β (PDGFR-β, PDB ID: 3MJG [21]) and hypoxia-inducible factor-1α (HIF-1α, PDB ID: 4ZPR [22]) were downloaded from RCSB Protein Data Bank (http://www.pdb.org/ (accessed on 25 September 2021)). The Discovery Studio was used to embellish by removing the ligands, adding hydrogen, and removing water. Then, wogonin was docked with PDGFR-β and HIF-1α using the -CDOCKER method to evaluate their interaction energy and binding mode [23,24].

### 2.6. Cell Culture

Human pulmonary arterial smooth muscle cells (HPASMCs) were purchased from Cell Bioscience (Shanghai, China). HPASMCs were cultured in high-glucose DMEM containing 10% FBS and penicillin/streptomycin (1:100) and maintained in a humidified atmosphere of 5% CO_2_ in air at 37 °C. Before treatments, HPASMCs were cultured in 0.5% FBS medium overnight for starvation. When the experiments were conducted, cells were pretreated with wogonin (1, 3, and 10 μmol/L), bosentan (10 μmol/L) or vehicle (DMSO), followed by stimulation by PDGF-BB (40 ng/mL) for a further 48 h [25,26,27].

### 2.7. Cell Viability Assay

A CCK8 cell proliferation assay kit was used to measure cell viability according to the manufacturer’s instructions. CCK-8 (10 μL/well) was added to the medium for 3 h at 37 °C. The absorbance for each sample was assessed at 450 nm using a microplate reader. 

### 2.8. Immunofluorescence Staining

Proliferating cell nuclear antigen (PCNA) is a typical indicator of cell proliferation [28]. Vascular smooth muscle cells can regulate their own migration and that of vascular endothelial cells by secreting intercellular adhesion factors such as ICAM-1 and VCAM-1 [29]. Immunofluorescence analyses were performed to detect their respective protein levels using PCNA, VCAM-1, and ICAM-1 as primary antibodies, followed by Alexa Fluor 488 or Alexa Fluor 555-conjugated secondary antibody and visualized by a fluorescence microscope. The nuclei were visualized by Hoechst 33342 staining.

### 2.9. Wound-Healing Assay

Migration was evaluated by a wound-healing assay [30]. PASMCs were seeded in 96-well plates overnight. The cells were then scratched with sterilized tips and washed and changed to media without FBS for 24 h. After starvation and scraping the cell monolayer with a sterile micropipette tip, the cells were treated with PDGF-BB (40 ng/mL) or co-treated with wogonin and bosentan. The wound closure was digitally captured at 12, 24, and 36 h (magnification ×100). The area of cells grew that into the wound area was quantified and presented as wound confluence (%).

### 2.10. Western Blotting

The HPASMCs were washed with ice-cold PBS and harvested by scraping in a lysis buffer containing 20 mM Tris-HCl (pH 7.5), 150 mM NaCl, 1 mM Na_2_EDTA, 1 mM EGTA, 1% NP-40, 1% sodium deoxycholate, 2.5 mM sodium pyrophosphate, 1 mM beta-glycerophosphate, 1 mM Na_3_VO_4_, 1 µg/mL leupeptin, phosphatase inhibitor, and protease inhibitor. After lysis on ice for 30 min add the loading buffer, mix thoroughly and boil for 10 min in boiling water. Protein samples were separated by SDS-PAGE (8–10% polyacrylamide gels) and transferred to nitrocellulose membranes (Millipore, Billerica, MA) [31]. The primary antibodies against α-SMA (1:1000), calponin 1(1:1000), NOX4 (1:1000), and HIF-1α (1:1000) were used, with β-actin (1:2000) as an internal control. The primary antibody is incubated overnight and after appropriate secondary antibody incubation, the immunoblot on the membrane is developed by an enhanced chemiluminescence (ECL) system.

### 2.11. Statistical Analysis

The mean ± SEM was used to describe the data. For statistical analysis, the data were analyzed using GraphPad Prism 6 (GraphPad Software; LaJoya, CA, USA). Statistical comparisons were made using one-way ANOVA followed by Dunnett’s post hoc test. Significance was defined as *p*-values < 0.05.

## 3. Results

### 3.1. PPI Analysis of Targets of Wogonin against PH

**Figure 2:**
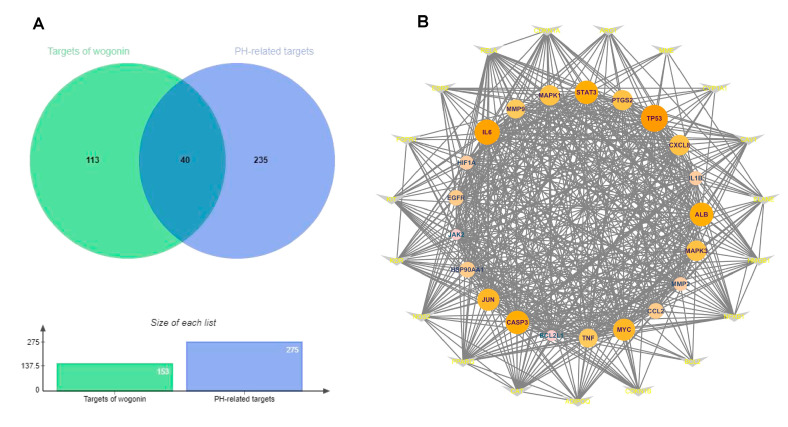
PPI network analysis of potential therapeutic targets of wogonin against PH. (**A**) The 40 intersection genes of wogonin and PH-related targets. (**B**) The PPI network of the 40 targets. the protein with higher degree was presented with larger node and darker color. The nodes in the inner circle are the core targets in the network. The color intensity of a node is proportional to the value of a degree in the network.

After removing the repeated targets from various databases, 113 targets of wogonin were collected. The 235 PH-related proteins and genes were identified using the Genecards database, the DisGeNET platform, and GenCLiP online tool. The 40 intersection genes of wogonin and PH-related proteins were considered to be potential therapeutic targets of wogonin against PH (Figure 2A, Table S1). The PPI network of potential anti-PH targets of wogonin was established, and the topology parameters were analyzed (Figure 2B). The targets with higher degree parameters in the network include: albumin (ALB), caspase-3 (CASP3), interleukin-6 (IL-6), signal transducer and activator of transcription-3 (STAT3), and tumor protein P53 (TP53).

### 3.2. Gene Ontology Analysis

**Figure 3:**
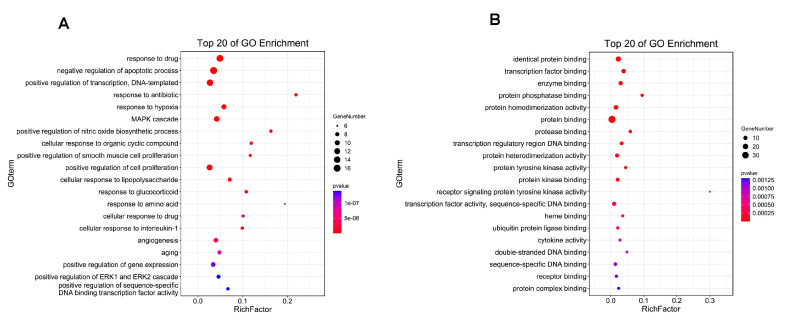
GO enrichment analysis. (**A**) biological process and (**B**) molecular function. The size of the dots indicated the number of enriched targets, and the color of the dots represented the degree of significance based on the *p*-value.

DAVID was used for the enrichment analysis of 40 potential targets of wogonin against PH. The BP and MF terms involved in wogonin were explored. The biological processes that wogonin may regulate include “negative regulation of apoptotic process”, “response to hypoxia”, “positive regulation of nitric oxide biosynthetic process”, “positive regulation of smooth muscle cell proliferation”, “positive regulation of cell proliferation”, and “cellular response to interleukin-1” (Figure 3A). The molecular functions involved in potential targets include “identical protein binding”, “transcription factor binding”, and “protein phosphatase binding”, etc. (Figure 3B).

### 3.3. Pathway Enrichment Analysis

**Figure 4:**
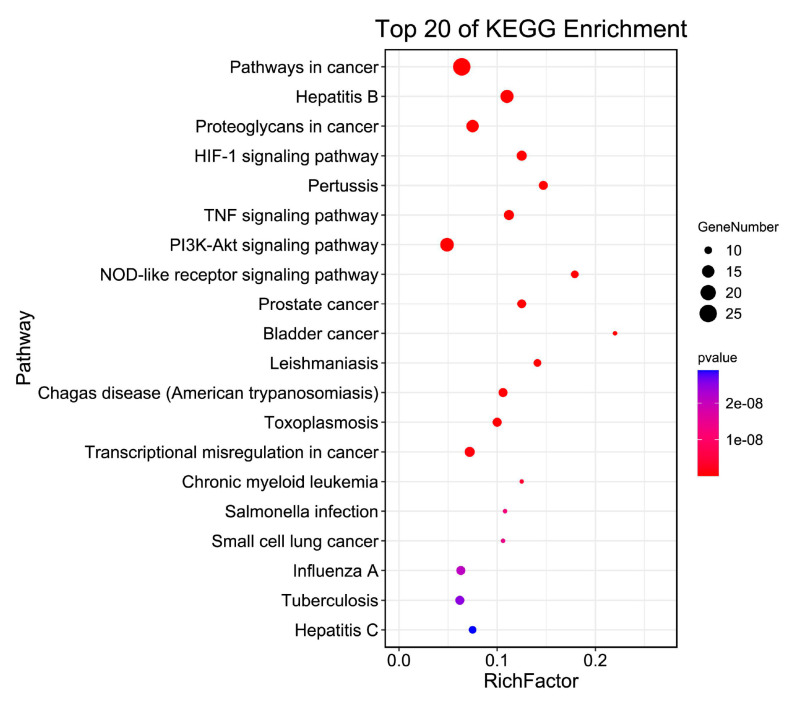
The KEGG analysis for the potential targets of wogonin against PH. The color represents the different *p*-values (<0.05), while the size of the circle represents the count.

The KEGG database was used for enrichment analysis. The top 20 pathways were screened. The results showed that the potential genes of wogonin against PH were mainly enriched in “HIF-1 signaling pathway”, “TNF signaling pathway”, and “PI3K-Akt signaling pathway” et al. (Figure 4).

### 3.4. Molecular Docking

**Figure 5:**
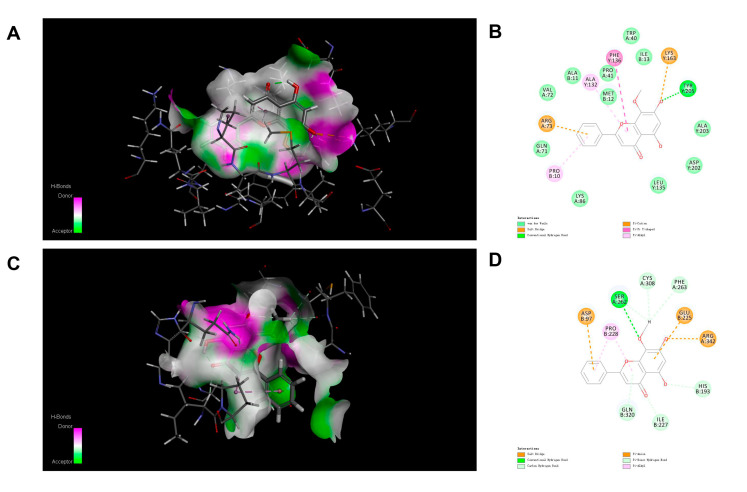
Molecular docking diagram. The 3D (**A**) and 2D (**B**) patterns of PDGFR protein binding with wogonin. The 3D (**C**) and 2D (**D**) patterns of HIF-1α protein binding with wogonin.

The structure of PDGFR and HIF-1α were taken from the PDB database. The binding mode of wogonin in the active site of PDGFR is represented in Figure 5A,B. Wogonin showed a conventional hydrogen bond with TYR 205 and a salt bridge with LYS 163, Other interactions including Pi–cation, Pi–Pi T-shaped, and Pi–alkyl were connected with ARG 73, PHE 136, ALA 132, and PRO 10. In addition, the binding mode of wogonin in the active site of HIF-1α is shown in Figure 5C,D. There were different types of hydrogen bonds with SER 262, CYS 308, PHE 263, GLN 320, ILE 227, and HIS 193. Other interactions including Pi–anion and Pi–alkyl were connected with ASP 97 and PRO 288, etc. The -CDCOKER ENERGY of wogonin with PDGFR and HIF-1α was 35.9576 and 20.8013 (Table 1). Wogonin showed good docking performance with both PDGFR and HIF-1α.

### 3.5. Wogonin Inhibits PDGF-BB-Mediated Proliferation of PASMCs

**Figure 6:**
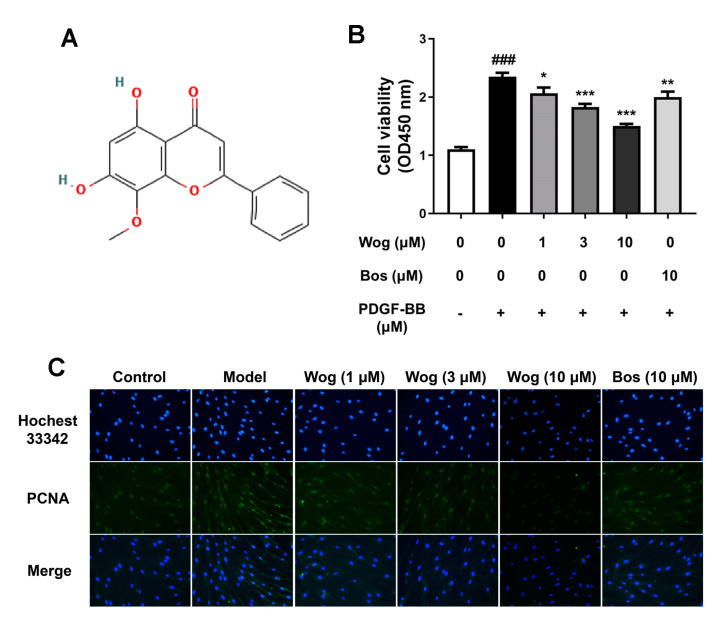
Wogonin inhibits PDGF-BB-mediated proliferation of PASMCs. PASMCs were cultured without or with PDGF-BB (40 ng/mL), PDGF-BB + wogonin (1, 3, 10 μM), PDGF-BB + bosentan (10 μM). (**A**) Chemical structure of wogonin. (**B**) Wogonin dose-dependently (1, 3, 10 μM) inhibits PDGF-BB-mediated PASMCs cell viability. (**C**) Effect of wogonin on PCNA expression of PDGF-BB-mediated PASMCs (original magnification ×200). Results are presented as mean ± SEM (n = 6). ### *p* < 0.001 vs. control group, * *p* < 0.05, ** *p* < 0.01, *** *p* < 0.001 vs. model group.

In order to investigate the anti-proliferation effect of wogonin, the PASMCs were treated with PDGF-BB for 48 h with different concentrations of wogonin (1, 3, 10 μM). The results of cell viability are shown in Figure 6B. Compared with the control group, PDGF-BB (40 ng/mL) significantly promoted the proliferation of PASMCs, and wogonin decreased the proliferation of PASMCs in a dose-dependent manner (1, 3, 10 μM). PCNA is a typical indicator of cell proliferation. The immunofluorescence staining of PCNA in PASMCs was used to detect the expression of PCNA in PASMCs. As shown in Figure 6C, the specific fluorescence intensity of PCNA in the model group was higher than that in the control group, while the fluorescence intensity in the wogonin-treated group was reduced. The results further proved that wogonin could inhibit the PDGF-BB-mediated proliferation of PASMCs.

### 3.6. Wogonin Inhibits Migration of PASMCs

**Figure 7:**
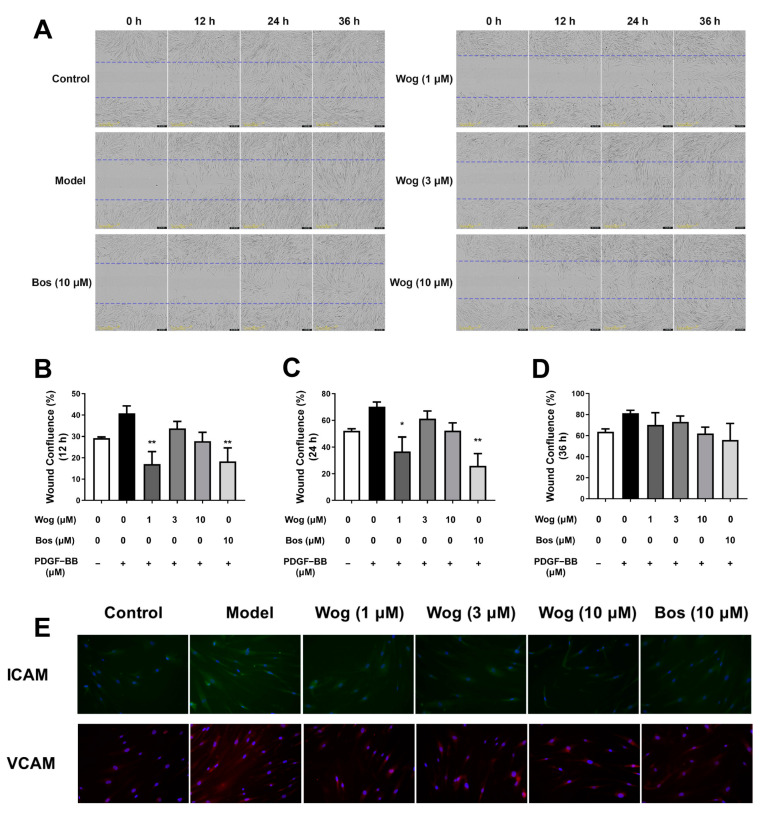
Wogonin inhibits PDGF-BB-mediated migration of PASMCs. (**A**) Microscopic image of wound healing experiment of PASMCs at 0, 12, 24, 36 h. (**B**) Wound confluence of PASMCs at 12 h (**B**), 24 h (**C**), and 36 h (**D**). (**E**) Effect of wogonin on ICAM and VCAM expression in PASMCs (original magnification ×200). Results are presented as mean ± SEM (n = 3). * *p* < 0.05, ** *p* < 0.01 vs. model group.

The effect of wogonin on PASMCs migration was detected by the wound healing experiment. The experimental result showed that wogonin (1 μM) significantly inhibited PDGF-BB-induced PASMCs migration after scratches were detected at 12 h and 24 h. To further verify the inhibitory effect of wogonin on PASMCs migration, the immunofluorescence in PASMCs was carried out to detect their protein levels of ICAM and VCAM. The results in Figure 7E showed that the fluorescence intensity of ICAM and VCAM in the PDGF-BB-induced model group was higher than that in the control group, while the fluorescence intensity of wogonin treated with different concentrations was lower than that in the model group (Figure 7E). Notably, the effect of wogonin on cell migration did not show a dose-dependent manner at the current concentration in the wound healing assay.

### 3.7. Effect of Wogonin on Phenotypic Switch of PASMCs

**Figure 8:**
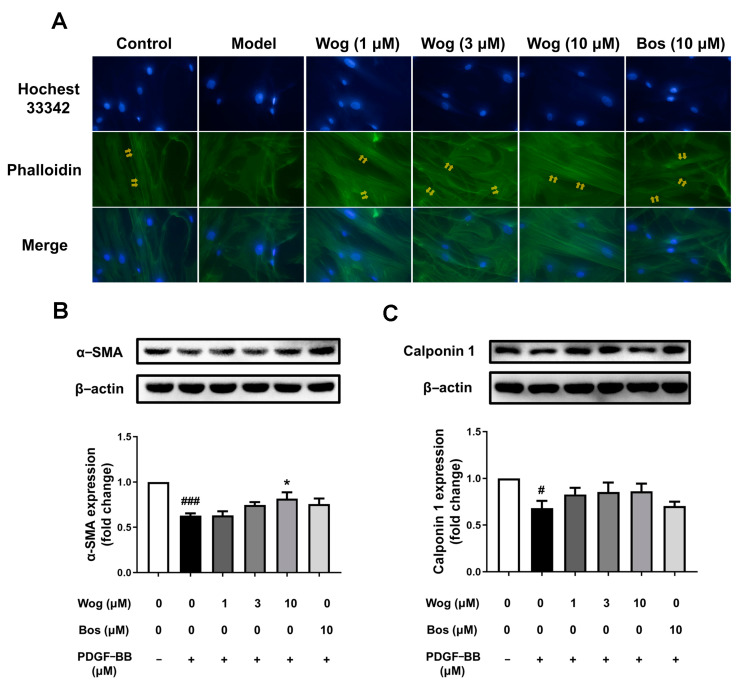
Effect of wogonin on phenotypic transformation of PASMCs. (**A**) The cytoskeleton is stained with phalloidin. (Original magnification ×400). Effect of wogonin on α-SMA (**B**) and calponin 1 (**C**) expression in PASMC by Western blot analysis. Results are presented as mean ± SEM (n = 3–4). # *p* < 0.05, ### *p* < 0.001 vs. control group, * *p* < 0.05 vs. model group.

The transformation of PASMCs from contractile/differentiated phenotype to synthetic/dedifferentiated phenotype can lead to vascular remodeling and promote the development of PH. Smooth muscle cells with contractile phenotype highly express α-SMA and calponin. Western blotting was used to detect the effects of wogonin on α-SMA and calponin expression. Compared with the control group, the expression of α-SMA and calponin in the model group was significantly decreased, while the expression of α-SMA was upregulated by wogonin (10 μM). The results suggest that PDGF-BB can promote the transformation of PASMCs from contractile to synthetic phenotype, while wogonin (10 μM) has the potential effect to inhibit this transformation (Figure 8B,C). We also observed the morphology and distribution of microfilaments in the cytoskeleton of PASMCs and found that wogonin could inhibit the phenotypic transformation to some extent (Figure 8A).

### 3.8. Effect of Wogonin on HIF-1/NOX4 Pathway

**Figure 9:**
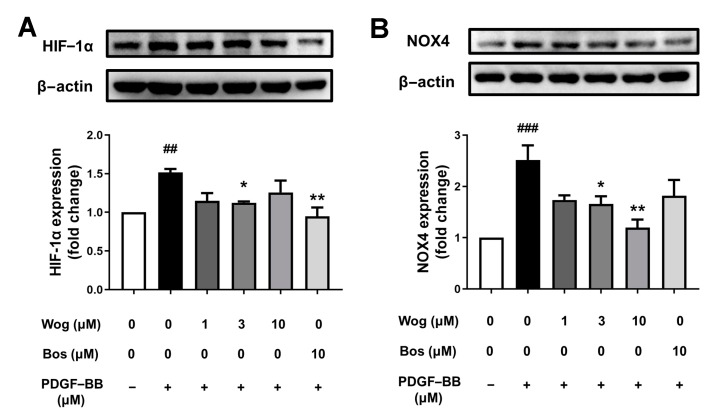
Effects of wogonin on HIF-1/NOX4 pathway. Effect of wogonin on HIF-1α (**A**) and NOX4 (**B**) expression in PASMCs by Western blot analysis. Results are presented as mean ± SEM (n = 3–4). ### *p* < 0.001, ## *p* < 0.01 vs. control group, * *p* < 0.05, ** *p* < 0.01vs. model group.

Hypoxia can promote the binding of HIF-1 to the hypoxia response element (HRE) in response to hypoxia status, while prolonged HIF-1 activation can lead to changes in pulmonary vascular structure. In addition to hypoxia stimulation, PDGF-BB can also induce HIF-1α in smooth muscle cells. The results confirmed that PDGF-BB can promote the expression of HIF-1α in smooth muscle cells, and the level of HIF-1α in the wogonin (3 μM) group was significantly decreased, indicating that wogonin can inhibit the expression of HIF-1α in PDGF-BB-induced PASMCs (Figure 9A). HIF-1α can bind to HRE on the NOX4 promoter, thus enhancing NOX4 promoter activity under hypoxia. Western blotting was used to analyze NOX4 expression in PDGF-BB-induced PASMCs. As shown in Figure 9, the expression of NOX4 was decreased by wogonin (3, 10 μM), while the inhibition of NOX4 expression was not significantly affected by wogonin (1 μM) (Figure 9B). The results showed that wogonin inhibited the expression of NOX4 in PASMCs, suggesting that the inhibitory effect of wogonin on PASMCs proliferation may be associated with inhibiting the HIF-1/NOX4 pathway.

## 4. Discussion

Wogonin, a flavonoid isolated from *Scutellaria baicalensis*, has attracted increasing scientific attention in recent years because of its potent activity on the cardiovascular system. The phenomenon of pulmonary vascular remodeling is often preceded by a progressive increase in vascular tone. Wogonin can inhibit aortic contraction induced by norepinephrine and potassium chloride in a dose-dependent manner by inhibiting extracellular calcium influx and intracellular calcium release [10]. On the basis of right ventricular pressure load caused by elevated pulmonary artery pressure, right ventricular remodeling and failure is one of the most important causes of mortality in patients with PH. Wogonin can delay the progression of ventricular hypertrophy and remodeling by specifically targeting Nrf-2 and promoting the expression of downstream antioxidant genes HO-1 and NQO-1 [11]. Hyperproliferation and decreased apoptosis of PASMC are the major triggers of media thickening in pulmonary vessels. In the present study, the results of network pharmacology indicated that targets of wogonin against PH were significantly enriched in processes related to smooth muscle cell proliferation and apoptosis (negative regulation of apoptotic process, positive regulation of smooth muscle cell proliferation, and positive regulation of cell proliferation). These findings are consistent with the results of biological experiments that wogonin can reduce the cell viability of PASMCs in a dose-dependent manner in PDGF-BB-induced proliferation. PASMCs can change from a highly differentiated contractile phenotype to a synthetic phenotype under specific external environmental changes such as injury, mechanical force, and oxidative stress [32,33]. Previous studies have shown that the transformation of PASMCs from contraction/differentiation phenotype to synthesis/dedifferentiation phenotype can lead to vascular remodeling and promote the occurrence of PH [34,35]. Partly based on the close relationship between pathological mechanisms and cell proliferation, PH has also been likened to cancer in the pulmonary circulation field. It is worth mentioning that the inhibitory proliferative effects of wogonin have also been demonstrated in a variety of tumor cells. Studies have shown that wogonin can negatively regulate HIF-1α and expression of monocarboxylate transporter-4 (MCT-4) to inhibit energy metabolism, cell proliferation, and angiogenesis in human gastric cancer cells (SGC-7901) and human lung adenocarcinoma cells (A549) [36]. Wogonin can also inhibit the proliferation process of skin epithelioid cancer cells partially by inhibiting Notch1 gene expression [37]. Wogonin also has a specific cytotoxic effect on lung cancer cells, and this effect is related to the activation of apoptosis and the production of ROS [38]. 

In addition to cell proliferation, cell migration and the production and degradation of the extracellular matrix are also the pathological basis of vascular structure changes. Vascular smooth muscle cells can also regulate the migration of self as well as vascular endothelial cells and induce the occurrence of vascular inflammation by secreting intercellular adhesion factors including ICAM and VCAM [39,40]. Wound healing assay results showed that wogonin could significantly inhibit PASMC migration and partially reduce ICAM and VCAM expression. Moreover, it is worth noting that wogonin exhibited an excellent inhibitory effect on cell migration in a variety of different kinds of cells. In vitro, migration of human alveolar adenocarcinoma A549 cells in the inflammatory microenvironment was found to be inhibited by wogonin (5, 10, 20 μM) [41]. Wogonin (15, 30, 60 μM) also inhibits migration and invasion of B16-F10 melanoma cells [42]. In addition, transendothelial migration of human breast cancer cells MDA-MB-231 could also be inhibited by wogonin (1, 10, 100 μM) [43]. Moreover, wogonin can inhibit H_2_O_2_-induced migration of HUVECs as well as microvessel sprouting from rat aortic rings by targeting the PI3K/Akt pathway and reducing VEGF expression [44]. In the nervous system, wogonin can inhibit the migration of microglial cells by downregulating the NF-κB pathway to ameliorate neuroinflammation during cerebral ischemic injury [45]. Wogonin inhibits melanoma cell migration, adhesion, invasion, and actin remodeling in vitro by suppressing the expression of matrix metalloproteinase-2 and Rac1 [42]. In the present study, we found that wogonin was effective in inhibiting PDGF-BB-induced proliferation of PASMCs at doses of 1, 3, and 10 μM. We note that at the same dose, it did not show a dose-dependent manner in the effect of wogonin on the migration of PASMCs. The reasons for this may include two points. For one, the IC50 of wogonin on cell migration is not in the range of doses that inhibit cell proliferation in this study. The dose range of wogonin, especially the nanomolar range, should be expanded in further experiments to explore more comprehensive and wider dose–effect curves. Secondly, although the wound healing assay is the classical method for evaluating the ability of cell migration, it still has drawbacks. For example, the influence of other biological processes is also present in the evaluation of migration capacity. The high concentrations of wogonin in this paper may have altered biological processes such as cell polarity, cytoskeletal remodeling, and thus ultimately the outcome of the cell migration experiments. The present study was mainly carried out to evaluate the proliferation of cells. Therefore, we will further investigate the effect of wogonin on the migration ability of PASMCs under micromolar and nanomolar dose ranges using the transwell chambers and real-time tracking of cell trajectories in further studies at a later stage. 

In the early stage of hypoxic stimulation, the change of intracellular oxygen content in PAECs causes PASMCs to undergo phenotypic transformation through a paracrine pathway and promotes angiogenesis to adapt to the hypoxic environment [46]. Under persistent hypoxic stimulation, contraction and pulmonary vascular structural reconstruction occur, which leads to an increase in pulmonary vascular resistance and pulmonary arterial pressure, resulting in the development of hypoxic pulmonary hypertension. This pathological phenotypic change may result from a combination of stress changes and other factors, such as oxidative stress, inflammation, and DNA damage [35]. The imbalance between proliferation and apoptosis of PASMCs is considered to be the main link in the occurrence of PH, in which HIF-1 plays an important intermediate role [47]. Hypoxia promotes HIF-1 binding to hypoxia response elements in response to the decrease in oxygen content, and prolonged HIF-1 activation causes structural changes in the pulmonary vasculature. In addition to hypoxic stimuli, PDGF can also induce HIF-1 α within smooth muscle cells [48,49]. After administration of wogonin, the level of intracellular HIF-1α decreased significantly. In addition, numerous studies have reported the effect of wogonin in downregulating HIF-1 expression in a variety of different cells. Wogonin can induce caspase-3 activation by inhibiting the expression of HIF-1 and survivin in EoL-1 cells, so as to improve the symptoms of chronic rhinosinusitis with nasal polyps [50]. Wogonin can also decrease HIF-1 expression by affecting its stability, and reduce the secretion of VEGF, thereby inhibiting tumor angiogenesis [51]. There is an interactive link between HIF-1 and NOX4. Studies have shown that HIF-1 can bind to the HRE at the NOX4 promoter in PASMCs cultured in vitro, which enhances the activity of the NOX4 promoter under hypoxia [52]. Besides, wogonin has an inhibitory effect on PDGF-induced smooth muscle cell proliferation [53]. Another structurally similar flavonoid, baicalin, was also shown to have the ability to inhibit PDGF-BB-induced proliferation of hepatic stellate cells [54]. Another research found that baicalin inhibited PDGF-BB-induced proliferation of vascular smooth muscle cells by inhibiting the PDGFRβ-ERK signaling pathway [55].

## 5. Conclusions

**Figure 10:**
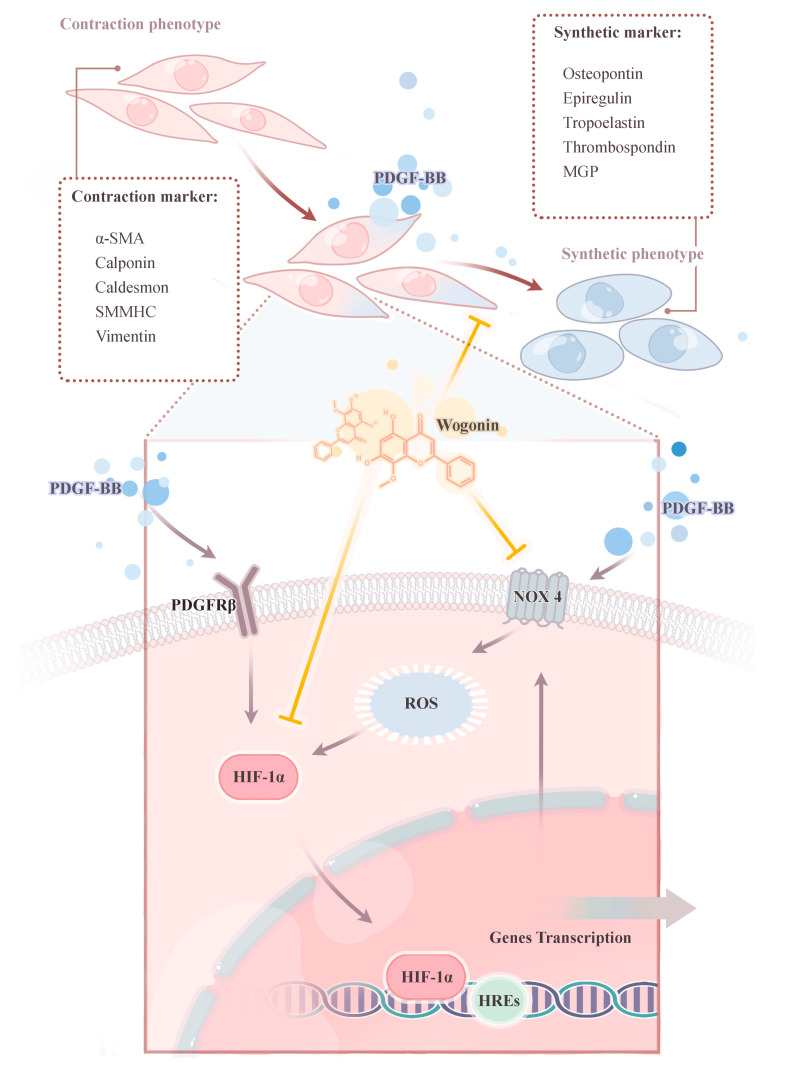
Schematic drawing showing the mechanisms of the anti-proliferative effects on PDGF-BB-induced proliferation of PASMCs.

In the present study, a combination of network pharmacology and experimental validation was applied to unveil the biochemistry basis and underlying mechanisms of wogonin in the treatment of PH. The results of network pharmacology shed light on the clinical and pharmaceutical significance of wogonin in treating PH. The effects of wogonin in inhibiting PASMCs proliferation, migration, and phenotypic switching are also consistent with the results of network pharmacology. Moreover, the anti-proliferative effect of wogonin may be mediated by the regulation of the HIF-1/NOX4 pathway (Figure 10). It is also worth noting that further experiments in vivo are required for their clinical translational potential in the future.

## Figures and Tables

**Table 1:** Molecular docking analysis of wogonin at active sites.

Compound	Target	-CDCOKER ENERGY
Wogonin	PDGFR	35.9576
	HIF-1α	20.8013

## Data Availability

The data used to support the findings of this study are available from the corresponding author upon request.

## Supplementary Materials

The following supporting information can be downloaded at: https://www.mdpi.com/article/10.3390/cimb45010037/s1, Table S1: The potential therapeutic targets of wogonin against PH.

## References

[1] Lau E.M.T., Giannoulatou E., Celermajer D.S., Humbert M. (2017). Epidemiology and treatment of pulmonary arterial hypertension. Nat. Rev. Cardiol..

[2] Archer S.L., Weir E.K., Wilkins M.R. (2010). Basic science of pulmonary arterial hypertension for clinicians: New concepts and experimental therapies. Circulation.

[3] Arora T.K., Arora A.K., Sachdeva M.K., Rajput S.K., Sharma A.K. (2018). Pulmonary hypertension: Molecular aspects of current therapeutic intervention and future direction. J. Cell. Physiol..

[4] Bello-Klein A., Mancardi D., da Rosa Araujo A.S., Schenkel P.C., de Lima Seolin B.G., Turck P. (2018). Role of Redox Homeostasis and Inflammation in the Pathogenesis of Pulmonary Arterial Hypertension. Curr. Med. Chem..

[5] Freund-Michel V., Guibert C., Dubois M., Courtois A., Marthan R., Savineau J.P., Muller B. (2013). Reactive oxygen species as therapeutic targets in pulmonary hypertension. Ther. Adv. Respir. Dis..

[6] Chen Y., Lu W., Yang K., Duan X., Li M., Chen X., Zhang J., Kuang M., Liu S., Wu X. (2020). Tetramethylpyrazine: A promising drug for the treatment of pulmonary hypertension. Br. J. Pharmacol..

[7] Maarman G.J. (2017). Natural Antioxidants as Potential Therapy, and a Promising Role for Melatonin Against Pulmonary Hypertension. Adv. Exp. Med. Biol..

[8] Wang J., Lu W., Wang W., Zhang N., Wu H., Liu C., Chen X., Chen Y., Chen Y., Jiang Q. (2013). Promising therapeutic effects of sodium tanshinone IIA sulfonate towards pulmonary arterial hypertension in patients. J. Thorac. Dis..

[9] Khan S., Kamal M.A. (2019). Can Wogonin be Used in Controlling Diabetic Cardiomyopathy?. Curr. Pharm. Des..

[10] Qu J.T., Zhang D.X., Liu F., Mao H.P., Ma Y.K., Yang Y., Li C.X., Qiu L.Z., Geng X., Zhang J.M. (2015). Vasodilatory Effect of Wogonin on the Rat Aorta and Its Mechanism Study. Biol. Pharm. Bull..

[11] Shi X., Zhang B., Chu Z., Han B., Zhang X., Huang P., Han J. (2021). Wogonin Inhibits Cardiac Hypertrophy by Activating Nrf-2-Mediated Antioxidant Responses. Cardiovasc. Ther..

[12] Wang X., Shen Y., Wang S., Li S., Zhang W., Liu X., Lai L., Pei J., Li H. (2017). PharmMapper 2017 update: A web server for potential drug target identification with a comprehensive target pharmacophore database. Nucleic Acids Res..

[13] Szklarczyk D., Santos A., von Mering C., Jensen L.J., Bork P., Kuhn M. (2016). STITCH 5: Augmenting protein-chemical interaction networks with tissue and affinity data. Nucleic Acids Res..

[14] Davis A.P., Grondin C.J., Johnson R.J., Sciaky D., Wiegers J., Wiegers T.C., Mattingly C.J. (2021). Comparative Toxicogenomics Database (CTD): Update 2021. Nucleic Acids Res..

[15] Daina A., Michielin O., Zoete V. (2019). SwissTargetPrediction: Updated data and new features for efficient prediction of protein targets of small molecules. Nucleic Acids Res..

[16] Fang S., Dong L., Liu L., Guo J., Zhao L., Zhang J., Bu D., Liu X., Huo P., Cao W. (2021). HERB: A high-throughput experiment- and reference-guided database of traditional Chinese medicine. Nucleic Acids Res..

[17] Stelzer G., Rosen N., Plaschkes I., Zimmerman S., Twik M., Fishilevich S., Stein T.I., Nudel R., Lieder I., Mazor Y. (2016). The GeneCards Suite: From Gene Data Mining to Disease Genome Sequence Analyses. Curr. Protoc. Bioinform..

[18] Piñero J., Ramírez-Anguita J.M., Saüch-Pitarch J., Ronzano F., Centeno E., Sanz F., Furlong L.I. (2020). The DisGeNET knowledge platform for disease genomics: 2019 update. Nucleic Acids Res..

[19] Wang J.H., Zhao L.F., Wang H.F., Wen Y.T., Jiang K.K., Mao X.M., Zhou Z.Y., Yao K.T., Geng Q.S., Guo D. (2020). GenCLiP 3: Mining human genes’ functions and regulatory networks from PubMed based on co-occurrences and natural language processing. Bioinformatics.

[20] Huang da W., Sherman B.T., Lempicki R.A. (2009). Systematic and integrative analysis of large gene lists using DAVID bioinformatics resources. Nat. Protoc..

[21] Rani U.P., Kesavan R., Ganugula R., Avaneesh T., Kumar U.P., Reddy G.B., Dixit M. (2013). Ellagic acid inhibits PDGF-BB-induced vascular smooth muscle cell proliferation and prevents atheroma formation in streptozotocin-induced diabetic rats. J. Nutr. Biochem..

[22] Zuo H.X., Jin Y., Wang Z., Li M.Y., Zhang Z.H., Wang J.Y., Xing Y., Ri M.H., Jin C.H., Xu G.H. (2020). Curcumol inhibits the expression of programmed cell death-ligand 1 through crosstalk between hypoxia-inducible factor-1alpha and STAT3 (T705) signaling pathways in hepatic cancer. J. Ethnopharmacol..

[23] Gagnon J.K., Law S.M., Brooks C.L. (2016). Flexible CDOCKER: Development and application of a pseudo-explicit structure-based docking method within CHARMM. J. Comput. Chem..

[24] Wu G., Robertson D.H., Brooks C.L., Vieth M. (2003). Detailed analysis of grid-based molecular docking: A case study of CDOCKER-A CHARMm-based MD docking algorithm. J. Comput. Chem..

[25] Kunichika N., Landsberg J.W., Yu Y., Kunichika H., Thistlethwaite P.A., Rubin L.J., Yuan J.X. (2004). Bosentan inhibits transient receptor potential channel expression in pulmonary vascular myocytes. Am. J. Respir. Crit. Care Med..

[26] Sysol J.R., Natarajan V., Machado R.F. (2016). PDGF induces SphK1 expression via Egr-1 to promote pulmonary artery smooth muscle cell proliferation. Am. J. Physiol. Cell Physiol..

[27] Yuan T., Zhang H., Chen Y., Jiao X., Xie P., Fang L., Du G. (2017). The Protective Effect of DL0805 Derivatives on Pulmonary Artery Cells and the Underlying Mechanisms Study. Curr. Vasc. Pharmacol..

[28] Dietrich D.R. (1993). Toxicological and pathological applications of proliferating cell nuclear antigen (PCNA), a novel endogenous marker for cell proliferation. Crit. Rev. Toxicol..

[29] Yusuf-Makagiansar H., Anderson M.E., Yakovleva T.V., Murray J.S., Siahaan T.J. (2002). Inhibition of LFA-1/ICAM-1 and VLA-4/VCAM-1 as a therapeutic approach to inflammation and autoimmune diseases. Med. Res. Rev..

[30] Grada A., Otero-Vinas M., Prieto-Castrillo F., Obagi Z., Falanga V. (2017). Research Techniques Made Simple: Analysis of Collective Cell Migration Using the Wound Healing Assay. J. Investig. Dermatol..

[31] Taylor S.C., Posch A. (2014). The design of a quantitative western blot experiment. Biomed Res Int.

[32] Badran A., Nasser S.A., Mesmar J., El-Yazbi A.F., Bitto A., Fardoun M.M., Baydoun E., Eid A.H. (2020). Reactive Oxygen Species: Modulators of Phenotypic Switch of Vascular Smooth Muscle Cells. Int. J. Mol. Sci..

[33] Zhang M.J., Zhou Y., Chen L., Wang Y.Q., Wang X., Pi Y., Gao C.Y., Li J.C., Zhang L.L. (2016). An overview of potential molecular mechanisms involved in VSMC phenotypic modulation. Histochem. Cell Biol..

[34] Maron B.A., Loscalzo J. (2013). Pulmonary hypertension: Pathophysiology and signaling pathways. Handb. Exp. Pharmacol..

[35] Tajsic T., Morrell N.W. (2011). Smooth muscle cell hypertrophy, proliferation, migration and apoptosis in pulmonary hypertension. Compr. Physiol..

[36] Wang S.J., Zhao J.K., Ren S., Sun W.W., Zhang W.J., Zhang J.N. (2019). Wogonin affects proliferation and the energy metabolism of SGC-7901 and A549 cells. Exp. Ther. Med..

[37] Xin N.J., Han M., Gao C., Fan T.T., Shi W. (2020). Wogonin suppresses proliferation and invasion of skin epithelioid carcinoma cells through Notch1. Cell. Mol. Biol..

[38] Wang C., Cui C. (2019). Inhibition of Lung Cancer Proliferation by Wogonin is Associated with Activation of Apoptosis and Generation of Reactive Oxygen Species. Balkan. Med. J..

[39] Li M., Zhu H., Hu X., Gao F., Hu X., Cui Y., Wei X., Xie C., Lv G., Zhao Y. (2021). TMEM98, a novel secretory protein, promotes endothelial cell adhesion as well as vascular smooth muscle cell proliferation and migration. Can. J. Physiol. Pharmacol..

[40] Xing J., Peng K., Cao W., Lian X., Wang Q., Wang X. (2013). Effects of total flavonoids from Dracocephalum moldavica on the proliferation, migration, and adhesion molecule expression of rat vascular smooth muscle cells induced by TNF-α. Pharm. Biol..

[41] Zhao Y., Yao J., Wu X.P., Zhao L., Zhou Y.X., Zhang Y., You Q.D., Guo Q.L., Lu N. (2015). Wogonin suppresses human alveolar adenocarcinoma cell A549 migration in inflammatory microenvironment by modulating the IL-6/STAT3 signaling pathway. Mol. Carcinog..

[42] Zhao K., Wei L., Hui H., Dai Q., You Q.D., Guo Q.L., Lu N. (2014). Wogonin suppresses melanoma cell B16-F10 invasion and migration by inhibiting Ras-medicated pathways. PLoS ONE.

[43] Song X., Zhou Y., Zhou M., Huang Y., Li Z., You Q., Lu N., Guo Q. (2015). Wogonin influences vascular permeability via Wnt/β-catenin pathway. Mol. Carcinog..

[44] Zhou M., Song X., Huang Y., Wei L., Li Z., You Q., Guo Q., Lu N. (2014). Wogonin inhibits H2O2-induced angiogenesis via suppressing PI3K/Akt/NF-κB signaling pathway. Vascul. Pharmacol..

[45] Piao H.Z., Choi I.Y., Park J.S., Kim H.S., Cheong J.H., Son K.H., Jeon S.J., Ko K.H., Kim W.K. (2008). Wogonin inhibits microglial cell migration via suppression of nuclear factor-kappa B activity. Int. Immunopharmacol..

[46] Dunham-Snary K.J., Wu D., Sykes E.A., Thakrar A., Parlow L.R., Mewburn J.D., Parlow J.L., Archer S.L. (2017). Hypoxic Pulmonary Vasoconstriction: From Molecular Mechanisms to Medicine. Chest.

[47] Shimoda L.A., Laurie S.S. (2014). HIF and pulmonary vascular responses to hypoxia. J. Appl. Physiol..

[48] Roos T.U., Heiss E.H., Schwaiberger A.V., Schachner D., Sroka I.M., Oberan T., Vollmar A.M., Dirsch V.M. (2011). Caffeic acid phenethyl ester inhibits PDGF-induced proliferation of vascular smooth muscle cells via activation of p38 MAPK, HIF-1α, and heme oxygenase-1. J. Nat. Prod..

[49] Xiao Y., Peng H., Hong C., Chen Z., Deng X., Wang A., Yang F., Yang L., Chen C., Qin X. (2017). PDGF Promotes the Warburg Effect in Pulmonary Arterial Smooth Muscle Cells via Activation of the PI3K/AKT/mTOR/HIF-1α Signaling Pathway. Cell. Physiol. Biochem..

[50] Khalmuratova R., Lee M., Mo J.H., Jung Y., Park J.W., Shin H.W. (2018). Wogonin attenuates nasal polyp formation by inducing eosinophil apoptosis through HIF-1α and survivin suppression. Sci. Rep..

[51] Song X., Yao J., Wang F., Zhou M., Zhou Y., Wang H., Wei L., Zhao L., Li Z., Lu N. (2013). Wogonin inhibits tumor angiogenesis via degradation of HIF-1α protein. Toxicol. Appl. Pharmacol..

[52] Diebold I., Petry A., Hess J., Gorlach A. (2010). The NADPH oxidase subunit NOX4 is a new target gene of the hypoxia-inducible factor-1. Mol. Biol. Cell.

[53] Huang H.C., Wang H.R., Hsieh L.M. (1994). Antiproliferative effect of baicalein, a flavonoid from a Chinese herb, on vascular smooth muscle cell. Eur. J. Pharmacol..

[54] Wu X., Zhi F., Lun W., Deng Q., Zhang W. (2018). Baicalin inhibits PDGF-BB-induced hepatic stellate cell proliferation, apoptosis, invasion, migration and activation via the miR-3595/ACSL4 axis. Int. J. Mol. Med..

[55] Dong L.H., Wen J.K., Miao S.B., Jia Z., Hu H.J., Sun R.H., Wu Y., Han M. (2010). Baicalin inhibits PDGF-BB-stimulated vascular smooth muscle cell proliferation through suppressing PDGFRbeta-ERK signaling and increase in p27 accumulation and prevents injury-induced neointimal hyperplasia. Cell Res..

