# Supplementary material for: Deciphering the Mechanism of Wogonin, a Natural Flavonoid, on the Proliferation of Pulmonary Arterial Smooth Muscle Cells by Integrating Network Pharmacology and In Vitro Validation
Source: Curr Issues Mol Biol. 2023 Jan 8;45(1):555–70. doi: 10.3390/cimb45010037 (PMC9858126; doi:10.3390/cimb45010037)
Supplement: Supplementary file 1 [file cimb-45-00037-s001.zip › cimb-2079898-supplementary.pdf]

Table S1 The potential therapeutic targets of wogonin against PH

Symbol	Name	Category	Gene Id
ADIPOQ	adiponectin, C1Q and collagen domain containing	Protein Coding	9370
ALB	albumin	Protein Coding	213
ARG1	arginase 1	Protein Coding	383
BCL2	BCL2 apoptosis regulator	Protein Coding	596
BCL2L1	BCL2 like 1	Protein Coding	598
CASP3	caspase 3	Protein Coding	836
CAT	catalase	Protein Coding	847
CAV1	caveolin 1	Protein Coding	857
CCL2	C-C motif chemokine ligand 2	Protein Coding	6347
CDKN1A	cyclin dependent kinase inhibitor 1A	Protein Coding	1026
CDKN1B	cyclin dependent kinase inhibitor 1B	Protein Coding	1027
CXCL8	C-X-C motif chemokine ligand 8	Protein Coding	3576
CYP1A1	cytochrome P450 family 1 subfamily A member 1	Protein Coding	1543
EGFR	epidermal growth factor receptor	Protein Coding	1956
ELANE	elastase, neutrophil expressed	Protein Coding	1991
ESR2	estrogen receptor 2	Protein Coding	2100
FGFR1	fibroblast growth factor receptor 1	Protein Coding	2260
HIF1A	hypoxia inducible factor 1 subunit alpha	Protein Coding	3091
HMGB1	high mobility group box 1	Protein Coding	3146
HSP90AA1	heat shock protein 90 alpha family class A member 1	Protein Coding	3320
IL1B	interleukin 1 beta	Protein	3553

		Coding	
IL6	interleukin 6	Protein Coding	3569
JAK2	Janus kinase 2	Protein Coding	3717
JUN	Jun proto-oncogene, AP-1 transcription factor subunit	Protein Coding	3725
KDR	kinase insert domain receptor	Protein Coding	3791
KIT	KIT proto-oncogene, receptor tyrosine kinase	Protein Coding	3815
MAPK1	mitogen-activated protein kinase 1	Protein Coding	5594
MAPK3	mitogen-activated protein kinase 3	Protein Coding	5595
MME	membrane metalloendopeptidase	Protein Coding	4311
MMP2	matrix metallopeptidase 2	Protein Coding	4313
MMP9	matrix metallopeptidase 9	Protein Coding	4318
MYC	MYC proto-oncogene, bHLH transcription factor	Protein Coding	4609
NFKB1	nuclear factor kappa B subunit 1	Protein Coding	4790
NOS2	nitric oxide synthase 2	Protein Coding	4843
PPARG	peroxisome proliferator activated receptor gamma	Protein Coding	5468
PTGS2	prostaglandin-endoperoxide synthase 2	Protein Coding	5743
RELA	RELA proto-oncogene, NF-kB subunit	Protein Coding	5970
STAT3	signal transducer and activator of transcription 3	Protein Coding	6774
TNF	tumor necrosis factor	Protein Coding	7124
TP53	tumor protein p53	Protein Coding	7157
